# MedChat: a fully offline multimodal AI system for privacy-preserving clinical anamnesis

**DOI:** 10.3389/frai.2026.1809142

**Published:** 2026-04-16

**Authors:** Jan Benedikt Ruhland, Doguhan Bahcivan, Jan-Peter Sowa, Ali Canbay, Dominik Heider

**Affiliations:** 1 Faculty of Mathematics and Natural Sciences, Heinrich Heine University Düsseldorf, Düsseldorf, Germany; 2 Department of Internal Medicine, University Hospital Knappschaftskrankenhaus Bochum, Bochum, Germany; 3 Department of Mathematics and Computer Science, Philipps University of Marburg, Marburg, Germany; 4 Institute of Medical Informatics, University of Münster, Münster, Germany

**Keywords:** AI in medicine, autoencoder, diffusion model, LLM, machine learning

## Abstract

Recent advances in large language models made it possible to achieve high conversational performance with substantially reduced computational demands, enabling practical on-site deployment in clinical environments. Such progress allows for local integration of AI systems that uphold strict data protection and patient privacy requirements, yet their secure implementation in medicine necessitates careful consideration of ethical, regulatory, and technical constraints. In this study, we introduce MedChat, a locally deployable virtual physician framework that integrates an LLM-based medical chatbot with a diffusion-driven avatar for automated and structured anamnesis. The chatbot was fine-tuned using a corpus of LLM-generated medical dialogues derived from publicly available symptom-disease datasets, enabling scalable and privacy-preserving training. A secure and isolated database interface was implemented to ensure complete separation between patient data and the model’s inference process. The avatar component was realized through a conditional diffusion model operating in latent space, trained on researcher video datasets and synchronized with mel-frequency audio features for realistic speech and facial animation. We demonstrate that the complete multimodal pipeline can operate fully offline on consumer-grade hardware while maintaining interactive response times (average latency: 2.9 ± 0.3 s) and stable system performance. Preliminary evaluation of generated dialogue indicates high linguistic coherence, supporting its suitability for structured anamnesis tasks. MedChat provides a privacy-preserving, resource-efficient, and multimodal solution for clinical data collection. While clinical validation is ongoing, the presented framework establishes a foundation for secure, locally deployable AI-assisted anamnesis in real-world healthcare settings.

## 1 Introduction

Artificial intelligence (AI) has reached a pivotal moment in its development, leading to transformative changes across nearly all sectors of human society (Jarrahi, 2018; Sarumi and Heider, 2024; Shi and Sun, 2024; Bengio et al., 2024). The rapid evolution of AI technologies presents both significant opportunities and substantial challenges, particularly with respect to adaptation, regulation, and ethical implementation. Central to this advancement is the emergence and continuous improvement of Large Language Models (LLMs), which are not only driving innovation in natural language processing and human-computer interaction but also in other fields (Sarumi and Heider, 2024; Alberts et al., 2023).

One prominent example is ChatGPT (Radford et al., 2018), an easily accessible and highly versatile LLM that has demonstrated utility across a wide range of applications. These include grammar correction, content refinement, application development, and even educational support. Despite their flexibility and impressive capabilities, such models possess significant risks. They may produce incorrect or misleading outputs, especially when their responses are accepted without critical scrutiny (Obradovich et al., 2024). Nevertheless, the societal potential of LLMs remains vast, particularly when appropriately integrated into domain-specific workflows.

In the field of medicine, AI occupies a uniquely sensitive and impactful position. The application of machine learning to medical data requires rigorous considerations due to the inherently private, complex, and ethically sensitive nature of healthcare information (Morley et al., 2020; Tajabadi et al., 2024). Ensuring data privacy, maintaining transparency, and adhering to ethical research practices are essential prerequisites that complicate the implementation of AI in clinical settings. However, despite these challenges, AI has already demonstrated its capacity to augment human expertise. For instance, convolutional neural networks have been reported to show superior performance in certain diagnostic tasks, particularly in medical imaging and pattern recognition (Haenssle et al., 2018; Choi et al., 2022).

One area where AI has the potential to further improve healthcare delivery is in anamnesis, the medical history-taking process. Effective anamnesis is complicated by the multivariate nature of symptoms, disease-specific manifestations, and the potential for asymptomatic progression, as seen in conditions such as COVID-19 (Gao et al., 2021). Traditional approaches often rely on standardized questionnaires, which may overlook critical individual nuances. In contrast, LLMs offer the flexibility to adapt questions dynamically based on patient responses, allowing for a more personalized and comprehensive data collection process.

By integrating LLMs into anamnesis workflows, clinicians could receive more structured, relevant, and individualized information, potentially leading to earlier and more accurate diagnoses. Much like the role of CNNs in medical image tasks, LLMs could serve as decision-support tools that assist healthcare professionals, reduce the risk of diagnostic errors, and contribute to the early identification of severe diseases. Thus, with appropriate safeguards, AI holds promise as a transformative aid in the practice of modern medicine.

As demonstrated in a recent study by Tu et al. (2025), LLMs have begun to outperform medical professionals in real-world clinical settings. Their model, the Articulate Medical Intelligence Explorer (AMIE), was evaluated in a controlled study where trained actors simulated patient interactions, allowing for direct comparison between AMIE and practicing clinicians during the anamnesis process. The objective of the study was to assess the viability of deploying a domain-specific LLM for clinical use, particularly in patient history-taking. The results revealed that AMIE, even when used by non-experts, outperformed licensed medical professionals across multiple diagnostic dimensions. This underscores the transformative potential of LLMs in supporting or even enhancing the clinical decision-making process, especially in initial diagnostic stages such as anamnesis.

Our study aimed to develop a locally deployable LLM, named MedChat, optimized for use in real-world clinical environments with constrained hardware capabilities. A key objective was to ensure secure integration with patient data systems. To that end, MedChat was designed to access a patient’s medical history only through a pre-processing interface: the relevant data is extracted and securely fed to the model prior to the interaction, without granting the LLM direct access to the underlying database. This approach balances the benefits of personalized context with stringent privacy and security standards. In addition, safeguards were implemented to mitigate risks associated with misuse or unauthorized manipulation of the chatbot, addressing a common concern in deploying AI systems in sensitive domains such as healthcare.

Beyond the development of the LLM itself, we also designed a human-machine interface to enhance the interaction quality and user engagement. To achieve this, video recordings of two researchers were used to train a diffusion model operating on latent space transformations, capable of generating synchronized speech and facial animations based on textual input. This enabled the creation of a virtual physician avatar, effectively simulating a human-like presence without requiring real-time human supervision. The resulting system offers a fully automated, responsive, and lifelike clinical interaction environment, potentially serving as a scalable solution for digital anamnesis.

The main contributions of this work are summarized as follows:

1. We present MedChat, a locally deployable large language model tailored for clinical anamnesis, capable of operating fully offline on consumer-grade hardware.
2. We integrate a diffusion-based virtual physician avatar for naturalistic, multimodal interaction combining speech and facial animation.
3. We employ parameter-efficient fine-tuning (LoRA) to achieve medical adaptation while maintaining the general-purpose capabilities of the base model.
4. We propose a secure database interface that enables structured, privacy-preserving integration of patient information without granting direct database access to the language model.
5. We demonstrate the complete end-to-end training and evaluation of all system components, laying the groundwork for future clinical validation.

### 1.1 Research in context

The integration of LLMs into healthcare has advanced rapidly, encompassing diverse applications ranging from diagnostic assistance and clinical documentation support to systems that approach partial or near-full autonomy in tasks traditionally performed by physicians, such as patient consultation and treatment planning (Tu et al., 2025; Xu et al., 2025). Current implementations include clinical decision support systems (Xu et al., 2025), symptom-based triage agents (Lu et al., 2024), and automated virtual health advisors (Lavan and Sangeetha, 2025). More experimental research has focused on developing fully autonomous “Virtual Doctor” systems capable of interacting directly with patients; however, these remain largely at the conceptual or prototype stage due to persistent ethical, regulatory, and safety challenges (Ruhland et al., 2025).

A major limitation in the deployment of LLMs and their embodied counterparts lies in their substantial computational requirements. High-performance models demand extensive memory resources and GPU processing capacity (Huang et al., 2024), often making cloud-based deployment necessary. While such infrastructure alleviates the need for on-premise hardware, it introduces significant concerns related to privacy, data protection, and compliance with medical data governance frameworks, particularly when sensitive information such as patient records, imaging data, or personal health histories is processed.

Most existing LLM-based healthcare systems rely on purely text-based chatbot interfaces that lack visual embodiment or avatar representation (Tu et al., 2025; Xu et al., 2025; Lu et al., 2024; Lavan and Sangeetha, 2025). Representative systems such as AMIE and MedAssist primarily focus on conversational reasoning and diagnostic support through text-only interaction, without incorporating multimodal output modalities such as visual avatars or speech-driven animation. Although these models provide robust functional capabilities, their absence of visual and emotional expressiveness can reduce patient engagement and trust, elements that are fundamental in clinical communication and adherence. To address this limitation, recent research has explored the integration of avatars and virtual agents capable of visual and nonverbal interaction (Winkler et al., 2025; DeTore et al., 2024). These systems aim to improve communication realism through facial expressions, gestures, and synchronized speech. Unlike avatars developed through game-engine-based real-time rendering or pre-recorded video playback, AI-driven avatar systems are typically implemented using generative approaches such as Generative Adversarial Networks (Goodfellow, 2014) or diffusion-based models (Ho et al., 2020, 2022). These architectures enable the synthesis of realistic facial expressions, gestures, and lip-synchronized speech, offering the potential for more natural and immersive interactions. However, such approaches introduce additional computational overhead and latency, particularly when high-fidelity generation is required in real time.

Despite these advancements, avatar-based or multimodal LLM systems continue to face substantial computational barriers. Systems such as AMIE and similar LLM-based assistants are primarily designed for cloud deployment, leveraging large-scale infrastructure to support high-performance inference. In contrast, avatar-based systems in the literature often prioritize visual realism and interaction quality, frequently relying on high-end GPUs or external rendering pipelines, while paying less attention to local deployability, data isolation, and regulatory constraints (Deutch et al., 2024; Yu, 2025). As a result, these systems are typically not optimized for constrained or privacy-sensitive clinical environments.

Consequently, current systems tend to optimize either conversational intelligence, visual fidelity, or deployment simplicity in isolation. They rarely address the combined constraints of local execution, multimodal synchronization, strict data isolation, and limited hardware resources. These trade-offs are often outsourced to cloud-based evaluations, creating a gap between experimental performance and real world clinical deployability.

Table 1 provides a structured comparison between MedChat and representative conversational medical AI systems, including AMIE (Tu et al., 2025), MedAssist (Xu et al., 2025), and other related approaches. The comparison is organized along key dimensions relevant to clinical deployment, including deployment architecture (cloud and local), presence of avatar-based multimodal interaction, privacy model, and stage of clinical readiness. As summarized in Table 1, existing systems generally lack the combination of fully local execution, multimodal avatar interaction, and strict data isolation implemented in MedChat.

**Table 1:** Comparison of existing LLM-based medical systems.

System	Deployment	Avatar interface	Privacy model	Clinical readiness
AMIE (Tu et al., 2025)	Cloud	No	Limited	Experimental
MedAssist (Xu et al., 2025)	Cloud	No	Partial	Prototype
HealsHealthAI (Lavan and Sangeetha, 2025)	Cloud	No	Partial	Research
MedChat	Local	Yes	Full isolation	Preclinical evaluation planned

In contrast to prior work, MedChat integrates a locally deployable LLM with a diffusion-based virtual physician avatar and a strictly isolated data processing pipeline. This design enables privacy-preserving operation without reliance on external servers while supporting multimodal interaction within a unified framework. By combining these elements, MedChat addresses a set of constraints that are typically treated separately in existing systems, thereby providing a more deployable solution for real-world clinical environments.

## 2 Materials and methods

### 2.1 Project overview

The primary objective of this project was to design and implement a locally deployable virtual physician system, referred to as MedChat, capable of operating entirely offline within clinical environments. The system is specifically developed to function without reliance on external servers or internet connectivity, thereby ensuring compliance with strict data protection regulations and maintaining the highest possible standards of patient privacy and information security.

The MedChat system is conceptualized as an interactive virtual physician embedded within a controlled physical environment. In a typical clinical workflow, the patient enters a private, designated consultation room equipped with a high-resolution display, camera, and computing interface. Within this space, the virtual physician is projected on the screen, enabling a naturalistic and confidential interaction. Prior to initiating the consultation, the patient is registered by clinical staff, and relevant portions of the patient’s medical history are securely preloaded into the system’s local database. This allows the virtual physician to access contextually relevant information during the anamnesis process while preserving data locality and avoiding external data transmission.

During the consultation, MedChat autonomously conducts a structured anamnesis, guiding the patient through a series of context-aware questions designed to capture essential clinical information. The collected data are then processed, organized, and securely stored in a local database using encrypted storage protocols. Following each session, the system automatically generates a structured summary report that synthesizes the key findings of the interaction. This report can subsequently be accessed and reviewed by licensed healthcare professionals, providing an efficient and standardized foundation for further clinical assessment and decision-making.

**Figure 1:**
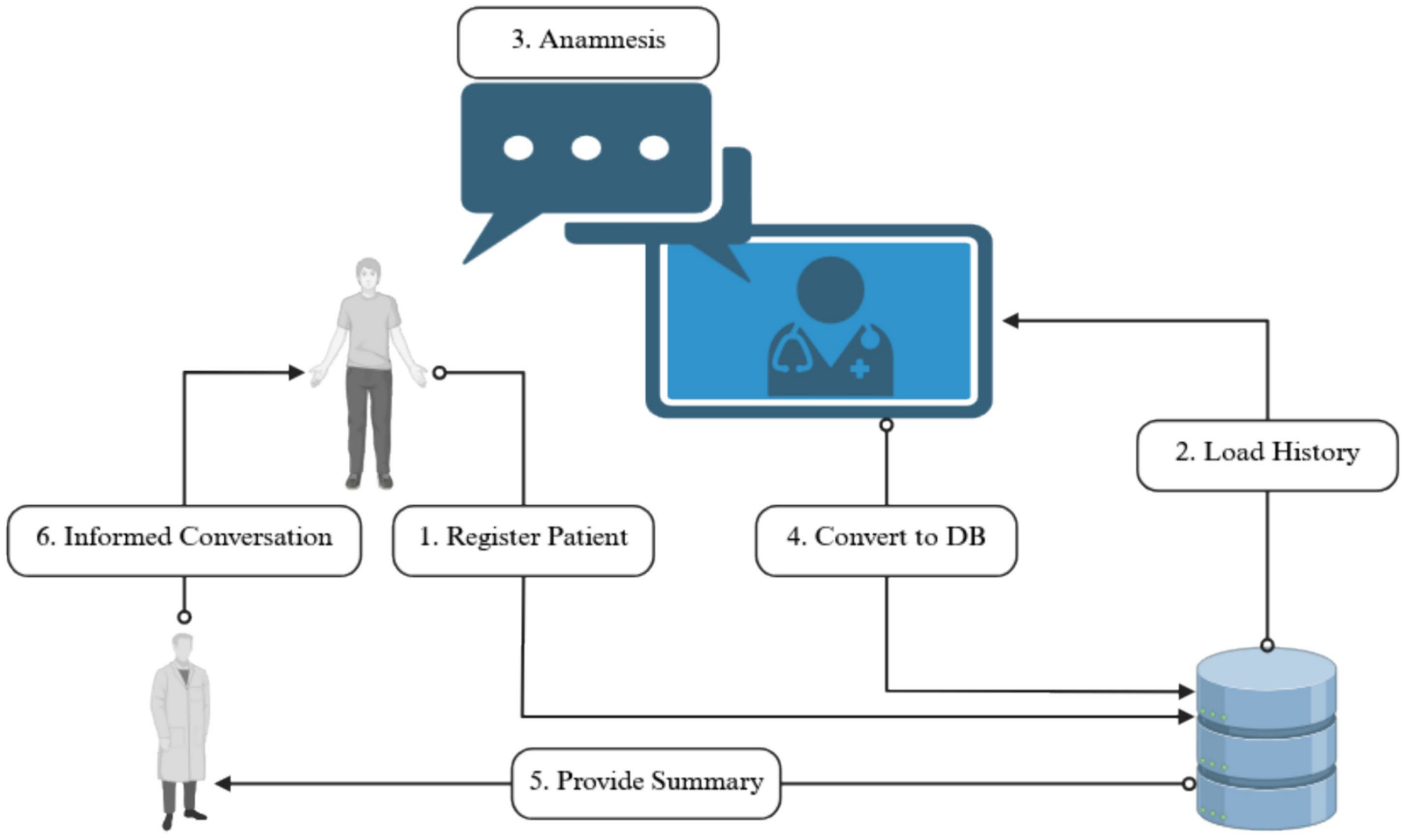
Overview of the anamnesis process facilitated by the MedChat system, illustrating its integration within standard clinical workflows. The image was created with https://www.biorender.com/.

Figure 1 presents a schematic overview of the anamnesis workflow facilitated by the MedChat system. By partially automating the patient intake procedure, MedChat contributes to the standardization of anamnesis across healthcare facilities, enhances the consistency of data collection, and optimizes the utilization of patient waiting times. Moreover, by reducing the administrative burden on medical personnel, the system allows healthcare providers to allocate more time to direct patient care and complex diagnostic reasoning. In this way, MedChat serves as both a technological innovation and a workflow enhancement tool, bridging artificial intelligence capabilities with the practical requirements of clinical practice.

While prior work has explored the deployment of large language models within medical contexts, the majority of existing systems rely on hybrid architectures that combine local components with cloud-based services for speech recognition, speech synthesis, avatar rendering, or model inference (Tu et al., 2025; Xu et al., 2025; Lu et al., 2024; Lavan and Sangeetha, 2025; Ruhland et al., 2025; Huang et al., 2024; Winkler et al., 2025; DeTore et al., 2024). In contrast, MedChat was explicitly designed as a fully self-contained system in which all computational components, including the language model, speech synthesis, audiovisual avatar generation, data storage, and security mechanisms, operate entirely offline on local hardware.

This design choice introduces substantial engineering challenges, particularly with respect to computational efficiency, memory constraints, and inference latency. Unlike cloud-based systems that can rely on elastic compute resources, MedChat must deliver interactive responsiveness under fixed hardware limits while maintaining clinically acceptable output quality. The system therefore required careful co-design of model architectures, inference strategies, and data pipelines to ensure real-time performance without external dependencies.

Several alternative architectural choices were considered during system design but deliberately excluded due to incompatibility with the target deployment constraints. Cloud-based inference pipelines and hybrid speech services were rejected despite their maturity, as they violate strict data locality requirements. Similarly, high-capacity diffusion or transformer-based video generation models were not adopted due to their prohibitive memory footprint and inference latency under local execution. These decisions highlight a central technical challenge addressed in this work: achieving acceptable multimodal interaction quality while operating under fixed computational budgets. The resulting architecture therefore represents a series of principled trade-offs rather than an attempt to maximize individual component performance.

### 2.2 The virtual doctor avatar

The virtual physician avatar in MedChat is not intended as a purely aesthetic addition but as a functional human–machine interface designed to support patient engagement, trust, and interaction flow during anamnesis Prior studies suggest that incorporating a visual, embodied representation into conversational agents can enhance user engagement and perceived empathy, particularly in sensitive healthcare interactions (Chattopadhyay et al., 2020). However, deploying embodied avatars in clinical settings introduces stringent technical constraints related to latency, audiovisual synchronization, and system reliability.

In this work, the avatar serves as a proof of concept for investigating whether high-fidelity, audiovisual physician representations can be generated locally and in real time without reliance on cloud-based rendering or streaming services. This requirement fundamentally shapes the system architecture and motivates several of the technical design choices described below.

MedChat uses a speech-to-text pipeline to transcribe patient speech in real time. Turn-taking is managed through a combination of voice activity detection and explicit cue words. Instead of reacting purely to silence, the system scans for command-like signals such as “continue” to initiate the next chatbot question. This approach prevents premature interruptions in cases where a patient pauses to think mid-sentence while ensuring the dialogue progresses smoothly. While this design reduces the naturalness of spontaneous conversational timing, it prioritizes complete and accurate information collection, which is critical in clinical anamnesis.

The resulting dialogue control system allows the LLM and avatar to maintain interactive responsiveness while respecting clinical workflow constraints and ensuring that patient input is fully captured.

#### 2.2.1 Network design and preprocessing

We developed a comprehensive preprocessing pipeline to extract both audio and video frames from recorded materials. The audio signals were resampled to 22,050 Hz to enhance the fine-tuning performance of the text-to-speech (TTS) model. Prior to transformation, the recordings were downsampled to 16 kHz to ensure compatibility with the Wav2Vec model (Baevski et al., 2020), which was employed for automated transcription. Pretrained Wav2Vec models from (Grosman, 2021) were utilized to generate bilingual transcriptions in English and German. Subsequently, the audio data were converted into mel-frequency spectrograms using a window size of 1,024 and a hop length of 128, extracting 80 mel-frequency components to capture perceptually salient acoustic features.

The speech synthesis component was implemented using the Piper TTS framework (OHF-Voice, n.d.), which follows an adversarial variational design inspired by the HiFi-GAN (Kong et al., 2020) and VITS (Kim et al., 2021) architectures. The training objective combines several complementary loss terms: (i) an adversarial loss applied to a least-squares discriminator-generator pair, (ii) a feature (perceptual) loss that minimizes the L1 distance between intermediate feature maps of real and generated audio, thereby enhancing perceptual fidelity in the frequency domain, and (iii) a Kullback–Leibler (KL) divergence term that regularizes the latent variable distribution. Together, these losses encourage both realistic waveform synthesis and consistent latent space structure, yielding speech output that closely resembles the target speaker’s tone and prosody. For this purpose, we adapted the pretrained Thorsten Voice model (Hansen, 2023a) for German and the Amy model (Hansen, 2023b) for English, training both on voice recordings provided by two researchers. The resulting customized voices ensured natural and consistent speech output in both languages, closely resembling the speaker’s tone and prosody.

To efficiently encode visual information, we developed a custom autoencoder that compresses image data into a compact latent-space representation, thereby reducing the computational cost of subsequent video generation. The model architecture was inspired by the Stable Diffusion framework (Rombach et al., 2021) and trained on facial image datasets supplied by the two researchers with video recordings around 30 min yielding over 70,000 images each with corresponding audio signals. For initial experiments, we additionally employed the “Visual Historical Record of American High School Yearbooks” dataset (Ginosar et al., 2017) to assess model generalization. The latent space was constrained to the range [−1, 1] using a hyperbolic tangent activation function to enhance numerical stability. Although this constraint may distort direct decoding, it improves robustness to noise and supports high-quality image reconstruction. To mitigate potential distortion, an adaptive normalization layer (Ruhland et al., 2025) was integrated into the decoder to map the constrained latent space back into an unconstrained representation. Furthermore, noise augmentation was introduced into the latent space to simulate imperfections typical of diffusion processes, promoting resilience during image generation. The autoencoder was designed to operate independently of input resolution, maintaining proportional compression relative to the image dimensions. Details of the model architecture and experimental results supporting these design choices are provided in Appendix A.

For the video generation stage, a conditional diffusion model was implemented, conditioned jointly on the input image and the mel-frequency representation of the corresponding audio frame. This multimodal conditioning enables synchronized lip movement and facial dynamics consistent with the spoken content. The diffusion model adopts a U-Net backbone architecture augmented with cross-attention layers, conditional embeddings, and multi-head self-attention mechanisms, ensuring both temporal coherence across sequential frames and semantic consistency between visual and auditory modalities.

During inference, the model diverges from conventional diffusion frameworks that typically initiate the generation process from random Gaussian noise. Instead, it begins from a pre-diffused default image, which serves as a structured prior, substantially reducing the number of denoising steps required for image reconstruction. This modification results in a significant decrease in computational cost and inference latency while maintaining high visual fidelity in the synthesized video output.

Furthermore, the system was evaluated for its ability to generate multiple subsequent frames, from the same pre-diffused input. This approach enables parallel frame generation, potentially bridging the performance gap between diffusion-based synthesis and real-time video rendering. The resulting architecture demonstrates that, with appropriate conditioning and initialization strategies, diffusion models can achieve interactive responsiveness suitable for real-time avatar-based communication systems.

**Figure 2:**
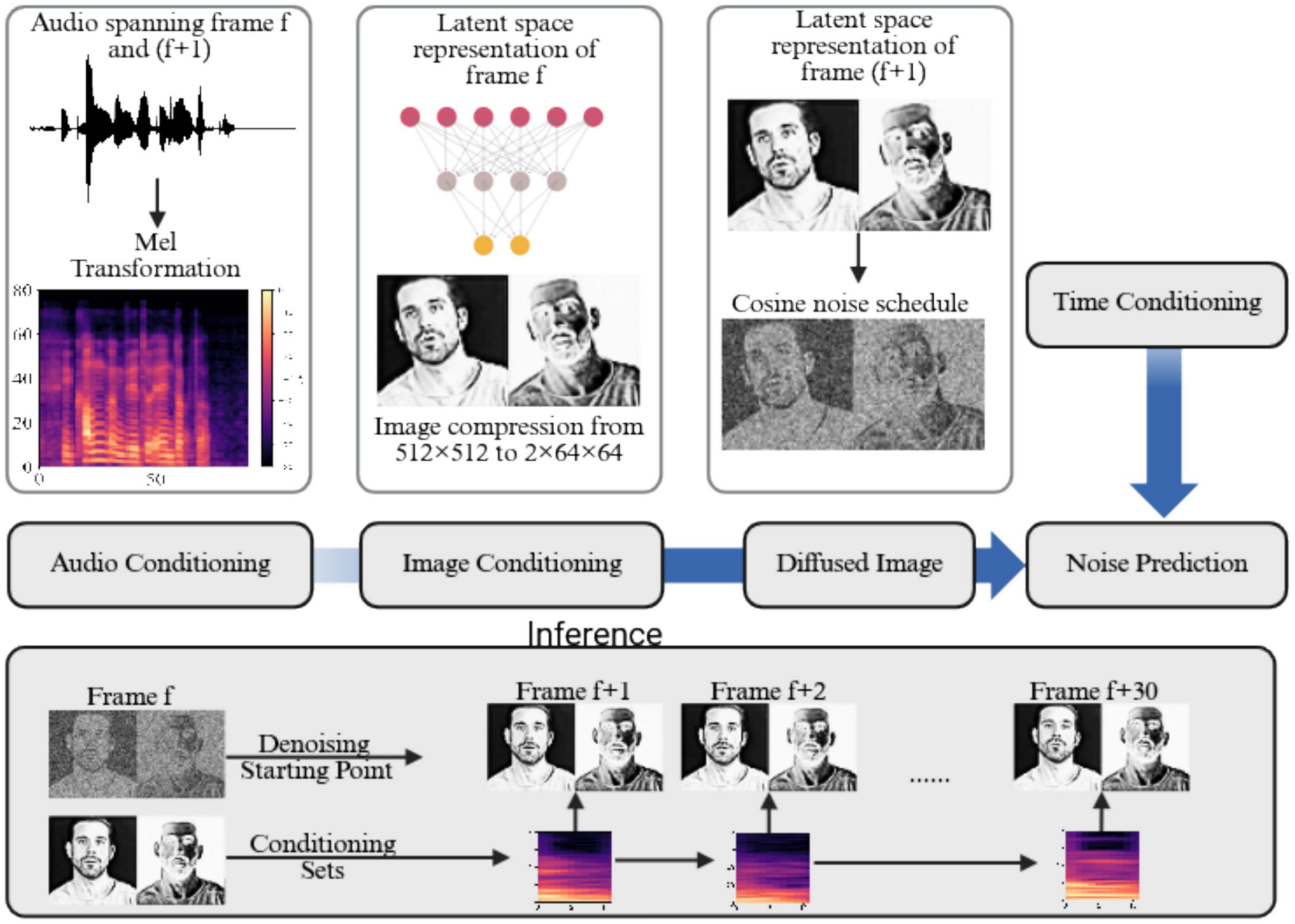
Illustration of the diffusion model training and inference pipeline for the virtual doctor avatar. During training, the model is conditioned on mel-frequency features, the previous frame, and the current time step from the cosine noise scheduler. The target frame is progressively diffused and used to train the network to predict the added noise. During inference, the trained model generates 1 second of video (30 frames) using a consistent conditioning image and a weakly diffused version of that image, while the conditioning mel-frequency inputs vary over time to reflect the changing audio features. The image was created with https://www.biorender.com/.

Figure 2 illustrates the training process of the diffusion model, highlighting the key modifications introduced during inference. During training, the model receives as input the current frame, the mel-frequency representation of the corresponding audio, and the previous frame, in addition to the timestep provided by the cosine noise scheduler. The target frame is progressively diffused, and the network is trained to predict the added noise, enabling it to learn the denoising dynamics. During inference, the trained model generates a sequence of frames, corresponding to 1 second of video (30 frames), by conditioning on a fixed reference image and a weakly diffused version of the same image. The mel-frequency inputs are updated continuously to reflect the evolving audio, allowing the system to produce temporally coherent video frames that are synchronized with the speech signal. This approach ensures both high-fidelity reconstruction and real-time responsiveness in the Virtual Doctor Avatar.

#### 2.2.2 Training procedure

Having established the preprocessing and network design, we next detail the fine-tuning procedures applied to each component, focusing on training stability, convergence behavior, and computational efficiency. The training process commenced with the fine-tuning of the audio data using the Piper TTS model. A reduced learning rate of 0.00001 was applied to both the generator and discriminator components to promote stable convergence and prevent overfitting. Training was conducted over 135 epochs, with model checkpoints evaluated every 50 epochs to assess qualitative and quantitative improvements. After 135 epochs, the synthesized voice demonstrated a high degree of perceptual similarity to the target speaker. However, additional training beyond this point led to a gradual degradation in voice quality, suggesting the onset of overfitting and confirming the optimal stopping point for fine-tuning.

Following the completion of the speech synthesis stage, training proceeded to the video generation component, utilizing the previously developed autoencoder as the foundational model for latent space compression. After verifying the model’s robustness on the public image dataset (Ginosar et al., 2017) (see Appendix A), the network was fine-tuned on recordings of the target researcher for 30 epochs. Training employed the Adam optimizer with an initial learning rate of 0.00001 and an L1 reconstruction loss as the objective function. A batch size of 32 was used, with all input images standardized to a resolution of 512 × 512 pixels. The autoencoder’s latent space was configured for a compression factor of 32.

For the diffusion-based generation stage, a cosine noise scheduling strategy (Nichol and Dhariwal, 2021) was employed to ensure smooth and stable convergence across 600 diffusion time steps. The model was trained for 1,000 epochs using the Adam optimizer with an initial learning rate of 0.0001, modulated by a cosine annealing schedule to facilitate adaptive step-size decay. The mean squared error (MSE) between the predicted and actual noise served as the training objective, consistent with standard Denoising Diffusion Probabilistic Model formulations (Rombach et al., 2021).

### 2.3 MedChat model development

#### 2.3.1 Datasets and conversation synthesis

To construct the primary dataset for large language model fine-tuning, we integrated three publicly available datasets from the Kaggle machine learning platform (Kaggle, 2024a; Kaggle, 2024b; Kaggle, 2024c). These datasets collectively comprised 10,080 samples, each containing a set of symptoms associated with a specific disease. These datasets are not derived from real patient-physician conversations but represent curated mappings of medical knowledge.

To transform the structured data into conversational training material, we employed a teacher-student knowledge distillation approach. The Meta-Llama-3.1-70B-Instruct (Dubey, 2024) was used as a teacher model to generate synthetic medical dialogues based on the symptom–disease pairs. These generated conversations were designed to reflect structured anamnesis interactions, including symptom exploration, follow-up questioning, and contextual clarification.

The resulting synthetic dialogue corpus was then used to fine-tune a smaller, deployment-oriented model (Meta-Llama-3.1-8B-Instruct), which forms the basis of the MedChat system. Preprocessing steps included formatting the dialogues into structured JSON files, removing non-informative or redundant responses (e.g., filler phrases), and standardizing the conversational structure to ensure consistency during training. This approach enables scalable generation of medically grounded conversational data while avoiding the need for sensitive clinical dialogue datasets, thereby supporting privacy-preserving model development and local deployment.

We leveraged the capabilities of the larger, pretrained teacher model to reduce training time and benefit from its existing contextual understanding. However, due to the substantial hardware requirements (approximately 140 GB of VRAM in 16-bit precision) the 70B model is impractical for deployment in typical medical facility settings. Hence, our final MedChat implementation is based on the 8B version, which is more feasible for real-world applications.

To ensure the generated dialogues were clinically meaningful and structurally consistent, we provided the teacher model with detailed generation guidelines. These guidelines, described in the Appendix B, included instructions on conversational tone, question structure, and appropriate responses to patient input. The model was allowed to generate up to 1,500 tokens per dialogue, enabling the creation of rich and informative medical conversations.

By repeating the generation process for every entry in the symptom-disease dataset, we created a large synthetic corpus of anamnesis dialogues. This synthetic dataset served as the foundation for fine-tuning our MedChat model, allowing it to effectively simulate medical interviews despite the absence of real patient interaction data.

Following the generation of 10,080 medical dialogues, we applied preprocessing steps to prepare the dialogues for model training, including formatting the conversations into structured JSON files. One common issue observed in the generated text was the excessive use of filler or acknowledgment phrases, e.g., “I see.” or “Noted.” To improve quality and coherence, we filtered out chatbot responses that did not contain a question and hence contained redundant information.

For training, 10% of the dataset was randomly withheld to serve as a validation set, enabling the assessment of overfitting and evaluation of model performance on an independent subset.

#### 2.3.2 MedChat training

We selected the Meta-Llama-3.1-8B-Instruct model (Dubey, 2024) as the base model for fine-tuning, due to the impracticality of training a large language model from scratch, which would require extensive computational resources. Released in July 2024, this model is among the first robust large language models to combine a 128,000-token context window with multilingual capabilities, all within a manageable size of 8 billion parameters. Its relatively compact architecture allows it to be deployed and fine-tuned on consumer-grade or mid-range data center GPUs.

Fine-tuning was performed on a cluster of six NVIDIA A100 GPUs, each equipped with 40 GB of VRAM. We employed model parallelism by distributing instances of the model across all GPUs, splitting the dataset accordingly, and synchronizing gradients across devices during training. This setup enabled efficient parallel training and supported rapid experimentation. The dataset was divided into a 90:10 train-test split, with model evaluation conducted at the end of each epoch.

We used the AdamW optimizer (Loshchilov and Hutter, 2019) with a weight decay of 0.01 and an initial learning rate of 0.000005, coupled with a linear learning rate decay schedule. Each training batch consisted of 4 samples, and gradient accumulation was performed every 16 steps before synchronization across GPUs. Each epoch included 420 steps per GPU, and training was conducted for 4 epochs, resulting in approximately 1,680 training steps per device. In aggregate, this yielded 10,080 steps across all devices, corresponding to full coverage of our dataset.

To further enhance training efficiency and preserve the model’s broad capabilities, we adopted a parameter-efficient fine-tuning approach using Low-Rank Adaptation (LoRA) (Hu, 2021). Since the model is required to retain general-purpose functionalities, such as open-domain conversation, classification, summarization, and multilingual support, we aimed to avoid catastrophic forgetting. In line with the recommendations from (Hu, 2021), we limited fine-tuning to lightweight adapter modules inserted into the Key and Value projection matrices of the transformer layers. This selective update approach provides a favorable trade-off between computational cost and fine-tuning effectiveness while maintaining the integrity of the pretrained model’s diverse skill set.

#### 2.3.3 Database integration

To manage and store data collected during interactions, we integrated an SQLite database into the MedChat system using SQLAlchemy, a Python-based Object-Relational Mapping framework. This architecture supports efficient and structured data handling, enabling seamless communication between the application and the underlying database layer.

Following the conclusion of each anamnesis session, MedChat produces a structured summary and classification of the dialogue in JSON format. This output is subsequently parsed, converted into application-level data objects, and persisted in the SQLite database. These operations (parsing, transformation, and data storage) are implemented outside the model itself and are managed by a dedicated service class, which functions as an intermediary between the model’s output and the database. Although modern language models support direct database access via tool calls (Dubey, 2024), we explicitly exclude this functionality to mitigate potential security risks and maintain strict control over data access.

To further safeguard the system against adversarial inputs, such as prompt injection and jailbreak attacks (Liu, 2024), we integrated Prompt Guard (Hugging Face, 2024) into the application. Prompt Guard is a security-focused classification model that evaluates incoming prompts and categorizes them as benign, prompt injection, or jailbreak attempts. By embedding Prompt Guard into the input validation pipeline, we establish a proactive security layer that filters malicious content before it reaches the language model. This approach enhances the safety, reliability, and integrity of MedChat, particularly in the context of healthcare, where the consequences of model misuse can be significant.

### 2.4 Medical safety and risk mitigation

Ensuring patient safety is a primary requirement when deploying AI-based systems in clinical environments. The MedChat framework is designed as a structured anamnesis support tool that operates within a human-in-the-loop (HITL) workflow, ensuring that all final medical decisions are made by licensed healthcare professionals.

To mitigate risks associated with LLM hallucinations and the omission of critical diagnostic information, MedChat incorporates multiple layers of safeguards. At the interaction level, the system is guided by carefully designed prompts that restrict the model from generating diagnostic conclusions or treatment recommendations. Instead, the model is instructed to focus exclusively on retrieving and structuring patient history. These constraints are applied consistently to both user inputs and model outputs to maintain controlled and goal-oriented dialogue behavior.

In addition, MedChat integrates rule-based monitoring mechanisms to identify potentially life-threatening symptoms, such as indications of self-harm. These mechanisms operate through predefined symptom dictionaries, keyword and phrase matching, and normalized text patterns that capture clinically relevant expressions within user inputs. When such patterns are detected, the system generates alerts that are surfaced to the supervising clinician, enabling immediate human assessment and intervention. This mechanism is designed to support early recognition of critical conditions without replacing clinical judgment.

By combining prompt-level constraints, rule-based safety monitoring, and continuous human supervision, MedChat is intended to function strictly as an assistive tool for structured anamnesis rather than an autonomous diagnostic system. This design aligns with its intended use as a decision-support application and reduces the risk associated with unsupervised medical decision-making.

From a regulatory perspective, such systems fall within the scope of medical decision-support tools. The current implementation is therefore positioned as a research prototype, and any future clinical deployment would require formal regulatory assessment, risk classification, and compliance with relevant certification and approval processes.

## 3 Results

### 3.1 Training of the autoencoder and diffusion model

**Figure 3:**
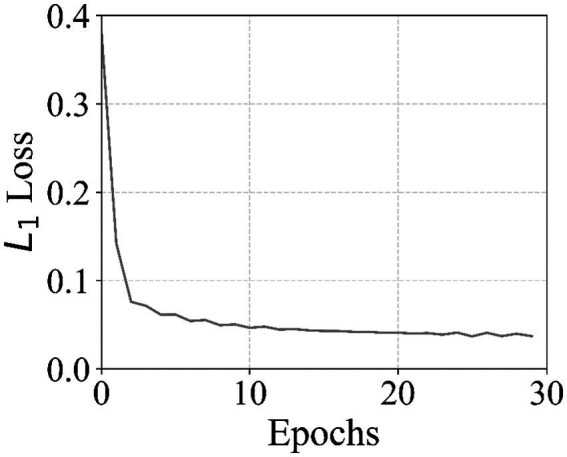
L1 reconstruction loss of the autoencoder during fine-tuning on recordings from a single researcher. The network was trained for 30 epochs with latent-space noise augmentation and a constrained latent representation. The curve demonstrates smooth and stable convergence, indicating effective learning of the input image reconstruction while maintaining latent-space regularity.

The fine-tuning of the autoencoders, designed with a constrained latent space and augmented with latent-space noise, demonstrated stable and consistent convergence throughout the training process. The autoencoder was personalized using recordings from a single researcher, enabling the network to adapt to specific facial and vocal characteristics. Figure 3 presents the L1 reconstruction loss over the course of 30 training epochs. At the beginning of training, the L1 loss started at approximately 0.382 and rapidly decreased during the first 10 epochs, reaching a value of 0.051. The subsequent epochs exhibited a slower, steady decline, ultimately stabilizing around 0.037 by the final epoch. This smooth convergence indicates that the network successfully learned to encode and reconstruct input images while maintaining the constraints imposed on the latent space. The latent-space noise augmentation further contributed to this stability by preventing overfitting and promoting robustness to perturbations in the latent representation. Notably, the short fine-tuning duration of 30 epochs was sufficient to achieve a low reconstruction error, demonstrating the efficiency of the proposed architecture and training regimen.

**Figure 4:**
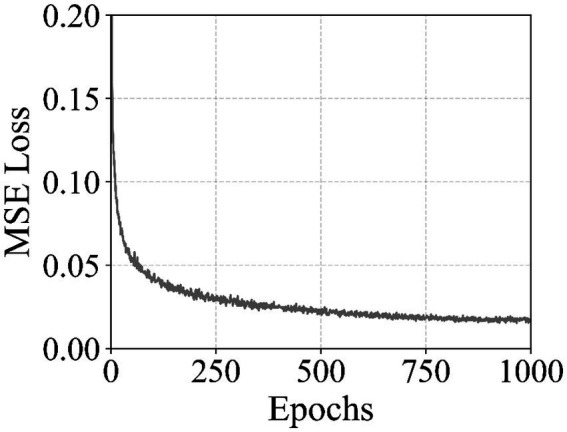
MSE value of the U-Net diffusion model during training over 1,000 epochs. The model predicts the noise added to latent representations conditioned on image and audio features using a cosine noise schedule. Minor fluctuations are observed due to the stochastic nature of the diffusion process, while the overall downward trend demonstrates stable convergence.

The U-Net model employed for diffusion-based video generation also exhibited clear convergence over its training regime. Figure 4 illustrates the MSE value between the predicted and applied noise across 1,000 epochs. The training curve begins at an initial MSE of approximately 0.774 and shows a consistent downward trend, reaching around 0.016 by the final epochs. While minor fluctuations of up to ±0.002 are observed throughout training, these are attributable to the stochastic nature of the diffusion process, where noise is randomly sampled at each timestep, and do not indicate instability. The consistent overall decrease in MSE demonstrates that the network effectively learned to denoise latent representations while conditioning on both image and audio features. These fluctuations also suggest that the model maintains adaptability to varying noise patterns, which is beneficial for generalization during inference.

The reported L1 reconstruction loss and mean squared error values are intended to characterize training stability and convergence behavior rather than to serve as absolute performance benchmarks. These metrics depend strongly on architectural choices, latent space dimensionality, noise schedules, and dataset characteristics, and are therefore not directly comparable across studies. In the context of this work, their primary purpose is to demonstrate that the proposed autoencoder and diffusion models can be trained reliably under constrained computational conditions and integrated into a real-time avatar pipeline without instability or collapse (see Table 2).

**Table 2:** Overview of model architectures and hyperparameters.

Component	Architecture	Loss function	Epochs	Learning rate
Autoencoder	Encoder-Decoder CNN	L1 reconstruction	30	0.00001
Diffusion U-net	Conditional U-net with cross-attention	MSE	1,000	0.0001
Piper TTS	Variational Inference GAN	Adversarial, L1, KL	135	0.00001
MedChat (LLM)	LLaMA 3.1-8B with LoRA	Cross-entropy	4	0.000005

### 3.2 Dialogue generation and MedChat fine-tuning

**Figure 5:**
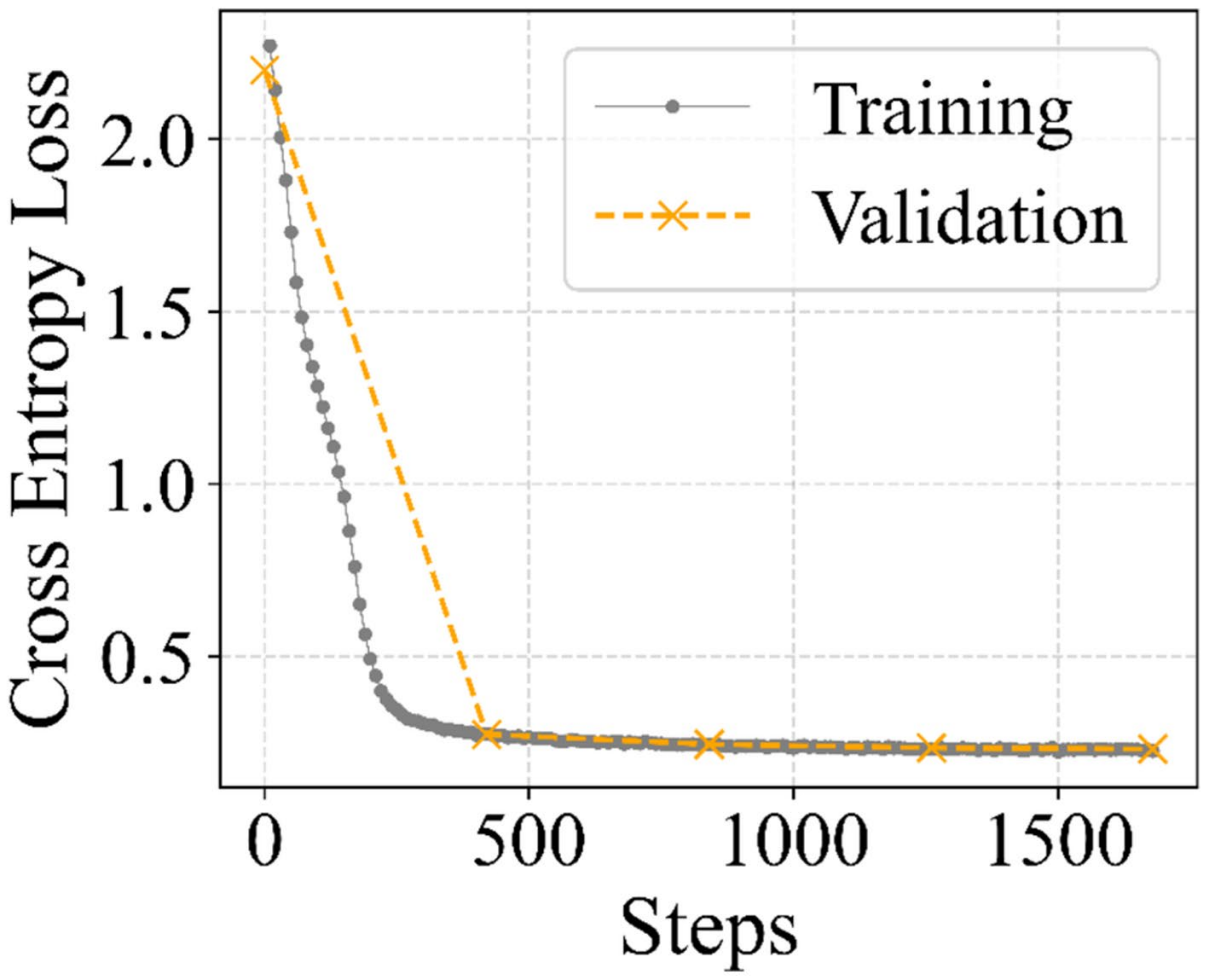
Training loss curves for the MedChat fine-tuning process. The model exhibits a rapid initial decrease in loss, followed by convergence, indicating effective learning and stable generalization to unseen data.

Figure 5 presents the fine-tuning dynamics of the MedChat model. At the onset of training, both the training and evaluation losses are relatively high, reflecting the model’s initial lack of adaptation to the specific task and substantial deviation from the desired output distributions. During the first 500 training steps, a rapid decrease in both losses is observed, indicating that the model quickly begins to capture relevant patterns from the training data. Beyond this point, the training loss continues to decrease gradually, while the evaluation loss stabilizes, demonstrating that the model maintains consistent performance on unseen data. The close alignment between training and evaluation loss throughout the remainder of the fine-tuning process suggests minimal overfitting and highlights the model’s ability to generalize effectively. Overall, these results indicate that the fine-tuning procedure successfully adapts MedChat to the target task while preserving robustness to novel inputs.

The simulated interaction described in Appendix C involves an initial consultation with a patient who has no prior history recorded in the MedChat system and for whom no external information from other models or sensor data is available. In this simulation, the patient is modeled as presenting symptoms consistent with pneumonia. The scenario is designed to evaluate the ability of MedChat to autonomously conduct a structured anamnesis, elicit relevant clinical information, and generate an initial patient summary entirely based on the interaction within the system. This setup provides a controlled environment for assessing the model’s performance in handling cases without any pre-existing contextual knowledge or external guidance.

From a deployment perspective, the full MedChat-Avatar system can operate on consumer-grade GPUs with as little as 40 GB of VRAM for end-to-end interaction, or on 24 GB of VRAM and only CPUs when the audio-only mode is used. This configuration substantially reduces hardware costs and energy consumption compared to cloud-based LLM solutions, which typically require 80–100 GB VRAM or distributed clusters for inference.

Table 3 provides a short summary of the models developed in the research as well as their training behavior.

**Table 3:** Quantitative summary of training performance across components.

Model	Initial loss	Final loss	Epochs	Convergence behavior
Autoencoder	0.382	0.037	30	Smooth convergence
Diffusion model	0.774	0.016	1,000	Stable with minor stochastic fluctuations
MedChat	2.45	0.23	4	Stable convergence without overfitting

### 3.3 System performance and latency

To provide a better understanding of the practical responsiveness of the MedChat framework, we measure the end-to-end latency of the interactive dialogue pipeline. End-to-end latency is defined as the time elapsed from the completion of the patient’s speech input to the initiation of the system’s avatar-generated response.

The processing pipeline consists of automatic speech recognition, LLM inference, text-to-speech synthesis, and diffusion-based avatar rendering. All measurements were conducted on a workstation equipped with an NVIDIA RTX 3090 GPU with 24 GB VRAM.

Across 100 simulated consultation sessions, the average end-to-end response latency was 2.9 ± 0.3 s. Among the individual components, diffusion-based avatar rendering accounted for the largest share of the total latency and represents the primary computational bottleneck. In contrast, LLM inference and TTS synthesis contributed a combined latency of approximately 0.7 s, while speech recognition introduced only minimal overhead. A detailed summary of the latency values can be observed in Table 4.

**Table 4:** Quantitative measurement of execution times.

Component	STT	LLM inference	TTS	Rendering
Mean execution time [s]	0.127 ± 0.012	0.699 ± 0.110	0.041 ± 0.025	1.806 ± 0.192

In addition to latency, system reliability was evaluated during prolonged continuous interaction sessions. The MedChat-Avatar system operated stably across all experiments, with no crashes, interruptions, or processing failures observed, indicating robustness under sustained use.

To provide an initial qualitative assessment of dialogue output, a random sample of 100 generated responses was reviewed by a medical professional. The sampled outputs were found to be grammatically coherent, contextually consistent, and appropriate for structured anamnesis. While this assessment does not constitute a formal clinical validation, it suggests that the system generates high-quality conversational outputs suitable for further evaluation in controlled clinical studies.

## 4 Discussion

The developed medical chatbot, MedChat, is designed as a clinical decision support tool rather than a stand-alone diagnostic system. All interactions require supervision by qualified medical personnel, and no direct diagnostic or therapeutic functions are performed autonomously. This design is essential for mitigating risks associated with large language models, including hallucinations and incomplete or inconsistent medical reasoning. To further enhance safety, MedChat incorporates mechanisms for detecting critical symptoms, alongside prompt-level safeguards that constrain both user inputs and model outputs. The system operates within a human-in-the-loop workflow, ensuring that all clinically relevant decisions remain under the supervision of qualified physicians. Within this framework, MedChat demonstrates robust technical performance under realistic hardware constraints, supporting the feasibility of fully offline deployment in clinical environments. The present work focuses primarily on the technical and methodological development of the MedChat framework. While the system generates fluent and structured dialogues for anamnesis, its clinical validity has not yet been formally established. A prospective evaluation study is currently being prepared in collaboration with clinicians at the University Hospital Bochum. This study will follow a human-in-the-loop design in which physicians assess AI-generated anamnesis dialogues using structured scoring criteria, including relevance, completeness, and usability. Until these results are available, MedChat should be interpreted as a proof-of-concept system for automated, structured patient interviews rather than a validated clinical tool.

From a technical perspective, the system demonstrates that multimodal AI components, including a large language model, text-to-speech synthesis, and a diffusion-based avatar, can be successfully integrated into a unified pipeline capable of real-time interaction on accessible hardware. The measured latency and stable runtime behavior indicate that natural conversational turn-taking is achievable without reliance on cloud infrastructure. This is particularly relevant for healthcare environments where data sovereignty and privacy are critical requirements.

Beyond technical performance, the interaction design introduces important human-centered considerations. In particular, the use of a generative avatar raises questions regarding user perception and psychological response. The “uncanny valley” phenomenon, where near-human representations evoke discomfort, may influence patient trust and engagement. Minor visual artifacts or inconsistencies in facial animation could be perceived as unnatural, especially in emotionally sensitive situations. Future evaluations will therefore include user experience assessments to better understand how avatar realism, expressiveness, and synchronization impact patient comfort, perceived empathy, and acceptance.

Several limitations of the current system must be acknowledged. First, the dialogue model is trained primarily on synthetically generated conversations derived from structured symptom datasets. While this enables scalable data generation, it may introduce biases and limit the model’s ability to generalize to complex or rare clinical scenarios. Second, the medical knowledge of the system is restricted to the information contained within its training corpus, which may not reflect the most recent clinical guidelines or emerging diseases. Third, the system has not yet been quantitatively evaluated for medical correctness, which remains a critical requirement for real-world deployment.

Additional limitations arise from the avatar generation process. The current implementation is based on a Markovian assumption, where each frame is conditioned only on the previous frame and the most recent audio segment. While this approach enables efficient real-time generation, it may lead to subtle temporal inconsistencies in facial motion. Future work will explore architectures incorporating longer temporal context, such as 3D convolutional models, to improve motion coherence and visual realism, albeit at increased computational cost.

Another important limitation concerns representation bias. The avatar model was trained on recordings from only two individuals, which may affect perceived empathy, authority, and relatability across diverse patient populations. Differences in age, gender, and cultural background are not currently represented, potentially limiting the system’s inclusivity. Expanding the training dataset to include a broader range of identities will be essential to ensure equitable and trustworthy interactions in clinical practice.

From a deployment perspective, MedChat is designed with scalability and maintainability in mind. The system operates on consumer-grade hardware, with the full multimodal pipeline running on GPUs with moderate memory requirements, while reduced configurations allow operation on lower-resource systems. The use of parameter-efficient fine-tuning techniques, such as Low-Rank Adaptation, enables efficient model updates without retraining the entire network. Furthermore, the modular architecture allows independent updates of system components, facilitating long-term maintenance and adaptation to evolving clinical requirements.

At the moment MedChat remains at a research prototype stage and is not intended for autonomous clinical decision-making. It has not yet undergone the regulatory processes required for deployment as a certified medical device. As such, it does not currently meet the requirements of frameworks such as the European Union Medical Device Regulation (MDR) for Software as a Medical Device (SaMD), nor the corresponding regulatory pathways defined by the U.S. Food and Drug Administration (FDA).

Transitioning such a system into a regulated clinical product would require adherence to established standards and processes, including ISO 13485 for quality management, ISO 14971 for risk management, IEC 62304 for medical software lifecycle development, and IEC 62366 for usability engineering. In addition, formal clinical validation studies and regulatory audits would be necessary to demonstrate safety, effectiveness, and compliance with applicable requirements prior to any clinical deployment.

In summary, MedChat demonstrates the feasibility of combining a locally deployable large language model with a diffusion-based avatar to enable structured, multi-modal anamnesis. While the system shows promising technical performance and interaction capabilities, further work is required to validate its clinical utility, improve generalization, and enhance user experience. The planned clinical evaluation, along with continued development of the underlying models and datasets, will be essential steps toward establishing MedChat as a reliable and practical tool for supporting healthcare professionals in real-world environments.

## 5 Conclusion

In this paper, we present a fully AI-driven anamnesis pipeline designed to autonomously conduct patient interviews and generate concise, structured summaries to assist physicians by highlighting key medical information. The system, referred to as MedChat, integrates language models optimized for local execution, enabling deployment on consumer-grade graphics hardware. This ensures accessibility and practicality for clinical environments with limited computational infrastructure.

MedChat was developed using structured medical datasets from publicly available sources, which were used to generate synthetic dialogue data for model training. However, it is important to note that the system has not yet undergone formal clinical validation. A prospective evaluation is planned at the University Hospital in Bochum, where the system’s utility will be assessed from two perspectives: the extent to which it supports medical professionals in their diagnostic workflow, and the degree of acceptance and trust exhibited by patients toward an AI-guided anamnesis process.

Future development efforts will focus on integrating more advanced LLMs and enhancing the visual and interactive quality of the system’s virtual avatar. A key ongoing challenge is maintaining a balance between system performance and practical feasibility. Many medical institutions operate under significant budget constraints and lack the resources required to deploy large-scale AI models. As such, our design prioritizes computational efficiency to offer a lightweight and cost-effective solution without compromising core functionality.

To promote transparency and foster further research in the field, we release both the synthetic training data and the developed neural network architectures as open-source software. This enables reproducibility of our results and provides a standardized benchmark for the development of future medical chatbot systems.

The system will be integrated into the Virtual Doctor project with testing and medical evaluation performed at the University Hospital Bochum in Germany. The results will be used to further improve on the framework and increase the benchmark dataset.

## Data Availability

The raw data supporting the conclusions of this article will be made available by the authors, without undue reservation.

## References

[ref1] Alberts I. L. Mercolli L. Pyka T. Prenosil G. Shi K. Rominger A. . (2023). Large language models (LLM) and ChatGPT: What will the impact on nuclear medicine be? Eur. J. Nucl. Med. Mol. Imaging 50, 1549–1552. doi: 10.1007/s00259-023-06172-w, 36892666 PMC9995718

[ref2] Baevski A. Zhou H. Mohamed A. Auli M. (2020). wav2vec 2.0: a framework for self-supervised learning of speech representations. arXiv.

[ref3] Bengio Y. Hinton G. Yao A. Song D. Abbeel P. Darrell T. . (2024). Managing extreme AI risks amid rapid progress. Science 384, 842–845. doi: 10.1126/science.adn0117, 38768279

[ref4] Chattopadhyay D. Ma T. Sharifi H. Martyn-Nemeth P. (2020). Computer-controlled virtual humans in patient-facing systems: systematic review and meta-analysis. J. Med. Internet Res. 22:e18839. doi: 10.2196/18839, 32729837 PMC7426801

[ref5] Choi Y. J. Park M. J. Ko Y. Soh M. S. Kim H. M. Kim C. H. . (2022). Artificial intelligence versus physicians on interpretation of printed ECG images: diagnostic performance of ST-elevation myocardial infarction on electrocardiography. Int. J. Cardiol. 363, 6–10. doi: 10.1016/j.ijcard.2022.06.012, 35691440

[ref6] DeTore N. Balogun O. Eberlin E. Dokholyan K. Rizzo A. Holt D. (2024). An artificial intelligence-based virtual human avatar application to assess the mental health of healthcare professionals: a validation study. J. Med. Extended Real. 1, 215–225. doi: 10.2196/preprints.58255 PMC13387219 42558081

[ref7] Deutch G. Gal R. Garibi D. Patashnik O. Cohen-Or D., (2024) TurboEdit: text-based image editing using few-step diffusion models

[ref8] Dubey A. (2024). The llama 3 herd of models. arXiv.

[ref9] Gao Z. Xu Y. Sun C. Wang X. Guo Y. Qiu S. . (2021). A systematic review of asymptomatic infections with COVID-19. J. Microbiol. Immunol. Infect. 54, 12–16. doi: 10.1016/j.jmii.2020.05.001, 32425996 PMC7227597

[ref10] Ginosar S. Rakelly K. Sachs S. Yin B. Efros A. A. (2017) A century of portraits: a visual historical record of American high school yearbooks. In: Proceedings of the IEEE International Conference on Computer Vision (ICCV) Workshops

[ref11] Goodfellow I. J. (2014). Generative adversarial networks. arXiv.

[ref12] Grosman J. (2021). Fine-Tuned XLSR-53 Large Model for Speech Recognition in German. Manhattan: Hugging Face.

[ref13] Haenssle H. A. Fink C. Schneiderbauer R. Toberer F. Buhl T. Blum A. . (2018). Man against machine: diagnostic performance of a deep learning convolutional neural network for dermoscopic melanoma recognition in comparison to 58 dermatologists. Ann. Oncol. 29, 1836–1842. doi: 10.1093/annonc/mdy166, 29846502

[ref14] Hansen M. (2023a), Thorsten medium checkpoint. Available online at: https://huggingface.co/datasets/rhasspy/piper-checkpoints/tree/main/de/de_DE/thorsten/medium (Accessed January 10, 2025).

[ref15] Hansen M. (2023b), Amy medium checkpoint. Available online at: https://huggingface.co/datasets/rhasspy/piper-checkpoints/tree/main/en/en_US/amy/medium (Accessed January 10, 2025).

[ref16] Ho J. Jain A. Abbeel P. (2020). Denoising diffusion probabilistic models. arXiv.

[ref17] Ho J. Salimans T. Gritsenko A. Chan W. Norouzi M. Fleet D. J. (2022). Video diffusion models. arXiv.

[ref18] Hu E. J. (2021). LoRA: low-rank adaptation of large language models. arXiv.

[ref19] Huang Y. Wan L. J. Ye H. Jha M. Wang J. Li Y. . (2024). Invited: new solutions on LLM acceleration, optimization, and application. In Proceedings of the 61st ACM/IEEE Design Automation Conference (DAC’24). Association for Computing Machinery

[ref20] Hugging Face (2024) meta-llama/Prompt-Guard-86M. Available online at: https://huggingface.co/meta-llama/Prompt-Guard-86M (Accessed October 29, 2024).

[ref21] Jarrahi M. H. (2018). Artificial intelligence and the future of work: human-AI symbiosis in organizational decision making. Bus. Horiz. 61, 577–586. doi: 10.1016/j.bushor.2018.03.007

[ref22] Kaggle (2024a). Disease Prediction Based on Symptoms. San Francisco: Kaggle.

[ref23] Kaggle (2024b). Disease Prediction Using Machine Learning. San Francisco: Kaggle.

[ref24] Kaggle (2024c). Disease and Symptoms Dataset. San Francisco: Kaggle.

[ref25] Kim J. Kong J. Son J. (2021). Conditional variational autoencoder with adversarial learning for end-to-end text-to-speech. arXiv.

[ref26] Kong J. Kim J. Bae J. (2020). HiFi-GAN: generative adversarial networks for efficient and high fidelity speech synthesis. arXiv.

[ref27] Lavan J. V. Sangeetha L. (2025). HealsHealthAI: Unveiling Personalized Healthcare Insights with Open Source Fine-Tuned LLM. Hoboken: Wiley.

[ref28] Liu Y. (2024). Prompt injection attack against LLM-integrated applications. arXiv.

[ref29] Loshchilov I. Hutter F. (2019). Decoupled weight decay regularization. arXiv.

[ref30] Lu M. Ho B. Ren D. Wang X. (2024). TriageAgent: Towards Better Multi-Agents Collaborations for large Language Model-Based Clinical Triage. Miami: Association for Computational Linguistics.

[ref31] Morley J. Machado C. C. V. Burr C. Cowls J. Joshi I. Taddeo M. . (2020). The ethics of AI in health care: a mapping review. Soc. Sci. Med. 260:113172. doi: 10.1016/j.socscimed.2020.113172, 32702587

[ref32] Nichol A. Dhariwal P. (2021). Improved denoising diffusion probabilistic models. arXiv.

[ref33] Obradovich N. Khalsa S. S. Khan W. Suh J. Perlis R. H. Ajilore O. . (2024). Opportunities and risks of large language models in psychiatry. NPP Digit. Psychiatry Neurosci. 2:8. doi: 10.1038/s44277-024-00010-z, 39554888 PMC11566298

[ref34] OHF-Voice. (n.d.). GitHub – OHF-Voice/piper1-gpl. Fast and local neural text-to-speech engine. GitHub repository. Available online at: https://github.com/OHF-Voice/piper1-gpl (Accessed April 3, 2026). GPL-3.0 license

[ref35] Radford A. Narasimhan K. Salimans T. Sutskever I. (2018). Improving Language Understanding by Generative Pre-Training. San Francisco: OpenAI Blog.

[ref36] Rombach R. Blattmann A. Lorenz D. Esser P. Ommer B. (2021). High-resolution image synthesis with latent diffusion models. arXiv.

[ref37] Ruhland J. B. Masoudian I. Heider D. (2025). Enhancing deep neural network training through learnable adaptive normalization. Knowl. Based Syst. 326:113968. doi: 10.1016/j.knosys.2025.113968

[ref38] Ruhland J. B. Wichmann J. Degtyar D. Martin R. Fehse L. Klau J. H. . (2025). The virtual doctor prescribing the future: diagnostics with interactive clinical decision support. Comput. Biol. Med. 196:110968. doi: 10.1016/j.compbiomed.2025.110968, 40839937

[ref39] Sarumi O. Heider D. (2024). Large language models and their applications in bioinformatics. Comput. Struct. Biotechnol. J. 23, 3498–3505. doi: 10.1016/j.csbj.2024.09.031, 39435343 PMC11493188

[ref40] Shi Y. Sun L. (2024). How generative AI is transforming journalism: development, application and ethics. J. Med. 5, 582–594. doi: 10.3390/journalmedia5020039

[ref41] Tajabadi M. Martin R. Heider D. (2024). Privacy-preserving decentralized learning methods for biomedical applications. Comput. Struct. Biotechnol. J. 23, 3281–3287. doi: 10.1016/j.csbj.2024.08.024, 39296807 PMC11408144

[ref42] Tu T. Schaekermann M. Palepu A. Saab K. Freyberg J. Tanno R. . (2025). Towards conversational diagnostic artificial intelligence. Nature 642, 442–450. doi: 10.1038/s41586-025-08866-7, 40205050 PMC12158756

[ref43] Winkler A. Kutschar P. Pitzer S. van der Zee-Neuen A. Kerner S. Osterbrink J. . (2025). Avatar and virtual agent-assisted telecare for patients in their homes: a scoping review. J. Telemed. Telecare 31, 207–221. doi: 10.1177/1357633X231174484, 37287248

[ref44] Xu R Shi W Wang J Zhou J Yang C. (2025). “MedAssist: LLM-empowered medical assistant for assisting the Scrutinization and comprehension of electronic health records.” In Companion Proceedings of the ACM on Web Conference 2025 (WWW'25). New York: Association for Computing Machinery 10.1145/3701716.3715186 PMC12995554 41858410

[ref45] Yu H. (2025). LLIA—enabling low-latency interactive avatars: real-time audio-driven portrait video generation with diffusion models. arXiv.

