# Supplementary material for: MedChat: a fully offline multimodal AI system for privacy-preserving clinical anamnesis
Source: Front Artif Intell. 2026 Apr 16;9:1809142. doi: 10.3389/frai.2026.1809142 (PMC13128582; doi:10.3389/frai.2026.1809142)
Supplement: Supplementary file 1 [file Data_Sheet_1.docx]

# Appendix A. Ablation Experiments

Our initial experiments were conducted using the selected dataset to design an autoencoder with a stable and well-constrained latent space, thereby improving training robustness during the subsequent diffusion modeling stage. To assess the impact of different latent space regularization strategies, we compared three autoencoder configurations based on the design described in table A1.

**Table A1:** Overview of the autoencoder architecture developed for this study. The model was implemented and tested across three configurations to investigate the influence of latent space regularization. In configuration 1, the tanh activation function at the encoder output was omitted. Configuration 2 integrated the reparameterization trick [33] to enforce a probabilistic latent distribution. Configuration 3, described in detail in this table, constitutes the final configuration adopted for all subsequent diffusion model experiments.

| **Module** | **Layer / Operation** | **Configuration** | **Output Resolution** | **Notes** |
| --- | --- | --- | --- | --- |
| **Encoder** | Adaptive Normalization | AdaptiveNorm((1, in_channels, 1, 1)) | N×N | Input normalization |
|  | Convolution | Conv2d(in_channels, 32, 3, 1, 1) | N×N | Initial feature extraction |
|  | ResBlock | ResBlock(32, 32) | N×N | Residual feature refinement |
|  | Downsampling | Conv2d(32, 32, 4, 2, 1) | N/2×N/2 | First spatial downsampling |
|  | ResBlock | ResBlock(32, 64) | N/2×N/2 | Feature expansion |
|  | Downsampling | Conv2d(64, 64, 4, 2, 1) | N/4×N/4 | Second spatial downsampling |
|  | ResBlock | ResBlock(64, 128) | N/4×N4 | Deep feature encoding |
|  | Downsampling | Conv2d(128, 128, 4, 2, 1) | N/8×N/8 | Latent compression |
|  | ResBlock | ResBlock(128, 128) | N/8×N/8 | High-level feature fusion |
|  | Projection | Conv2d(128, latent_channels, 3, 1, 1) | N/8×N/8 | Latent embedding |
|  | Activation | Tanh() | N/8×N/8 | Constrains latent space to [-1, 1] |
| **Decoder** | Adaptive Normalization | AdaptiveNorm((1, latent_channels, 1, 1)) | N/8×N/8 | Normalization of latent input |
|  | Linear Projection | Conv2d(latent_channels, 128, 1) | N/8×N/8 | Expands latent space into feature domain |
|  | ResBlock | ResBlock(128, 128) | N/8×N/8 | Residual learning in latent domain |
|  | Upsampling | ConvTranspose2d(128, 64, 4, 2, 1) | N/4×N/4 | First spatial upsampling |
|  | ResBlock | ResBlock(64, 64) | N/4×N/4 | Feature refinement |
|  | Upsampling | ConvTranspose2d(64, 32, 4, 2, 1) | N/2×N/2 | Second spatial upsampling |
|  | ResBlock | ResBlock(32, 32) | N/2×N/2 | Feature refinement |
|  | Upsampling | ConvTranspose2d(32, 16, 4, 2, 1) | N×N | Final spatial upsampling |
|  | ResBlock | ResBlock(16, 16) | N×N | Final feature smoothing |
|  | Output Convolution | Conv2d(16, out_channels, 3, 1, 1) | N×N | Image reconstruction |
|  | Activation | Tanh() | N×N | Constrains pixel values to [-1, 1] |

Our first approach was to establish a base line with an unconstrained latent space, i.e. where the latent variables were allowed to take arbitrary continuous values. The second approach was a variationally constrained latent space, where a normal prior distribution N(0,I) was enforced through the reparameterization trick and an additional Kullback-Leibler divergence loss term as described in [33]. The last approach contained a deterministically constrained latent space, where a hyperbolic tangent activation was applied to project latent variables into the range [-1,1]. An adaptive normalization layer [35] was incorporated in the decoder to re-establish consistent statistics during reconstruction.

The reparameterization trick introduces stochasticity into the latent representation while maintaining differentiability, enabling efficient gradient-based optimization. It can be expressed as:

(1)                                                             z=μ+σ×ϵ, ϵ∼N(0,I)

where μ and σ represent the mean and standard deviation predicted by the encoder, and ϵ denotes a noise sample drawn from a standard normal distribution. To regularize the latent space toward a standard normal prior, we employed the Kullback-Leibler divergence loss, defined as:

(2)                                                 L_KL_​ = -0.5 ∑^d^_i=1_​(1+log(σ_i_^2^​)-μ_i_^2​^-σ_i_^2^​)

where the sum is over the full dimensionality d of the latent space.

The reconstruction objective for all configurations was the L1 loss between the input and reconstructed image:

(3)                                                                 L_rec_​=∥x-x∥^1^_1_

The autoencoder models were trained for 30 epochs using images of 256×256 resolution. To enhance the generalization capability of the network, a set of geometric data augmentation techniques was applied, including random rotations, translations, scalings, and shear transformations. All transformations employed zero padding to preserve image dimensions. The training objective combined an L1 reconstruction loss, as defined in equation (3), with a Kullback-Leibler divergence term serving as a regularization component for configuration 2. The regularization coefficient was varied between 0.000001and 0.01 to assess its impact on latent space stability. Optimization was performed using the AdamW algorithm [39], which provides adaptive moment estimation with decoupled weight decay to improve convergence behavior.

Figure A6 presents the evolution of the L1 reconstruction loss across all three autoencoder configurations. The relatively weaker reconstruction performance observed in configuration 2 at a regularization weight of 0.01 can be attributed to the increased stochasticity introduced by the KL divergence, which induces variability in the decoding process. When the regularization coefficient was reduced to 0.000001, the reconstruction quality improved. However, the latent space exhibited poor constraint, with activation magnitudes exceeding an order of magnitude of 10. Balancing the KL and L1 loss terms proved challenging, as excessive regularization reduced reconstruction fidelity, whereas insufficient regularization led to latent instability. Consequently, configuration 3 was selected as the final model, as it achieved a well-defined latent representation suitable for subsequent latent space noise augmentation, thereby enhancing the robustness of the downstream diffusion model.

Figure A7 provides a qualitative comparison between the original input images and their corresponding reconstructions for each autoencoder configuration, illustrating the inherent trade-off between reconstruction fidelity and latent space regularity. The results for the strongly KL-regularized autoencoder are omitted, as training exhibited strong KL divergence dominance. Consequently, although the latent space distribution closely approximated a normal prior, the reconstruction quality degraded substantially. This behavior, consistent with the quantitative results shown in figure A6, indicates that excessive KL regularization overly constrains the latent representation, thereby impairing the network’s ability to accurately reconstruct fine-grained image details.


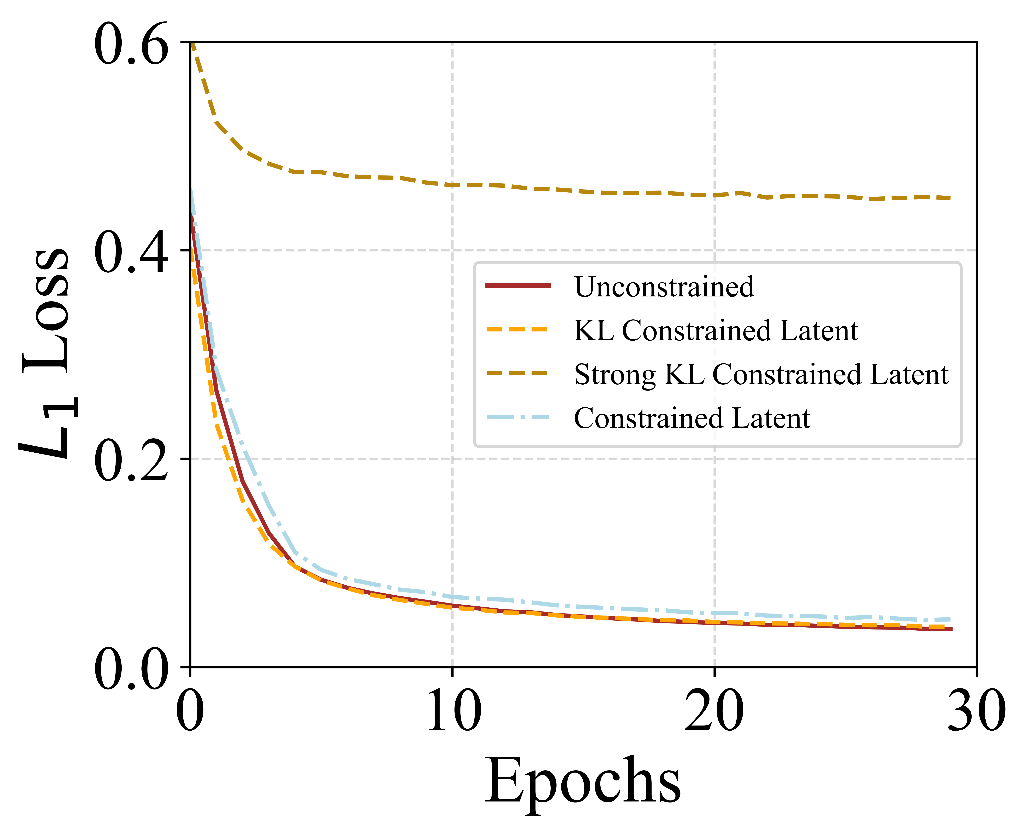
**Figure A6**: Training curves for the different autoencoder configurations. For configuration 2, which incorporates a KL divergence term, multiple regularization strengths were evaluated to assess the effect of relaxing the normal prior assumption. The curve labeled strong KL constraint corresponds to a regularization coefficient of 0.01, reflecting a strong enforcement of the prior, whereas the weak KL constraint (orange curve) employs a coefficient of 0.000001, allowing greater flexibility in the latent space representation.


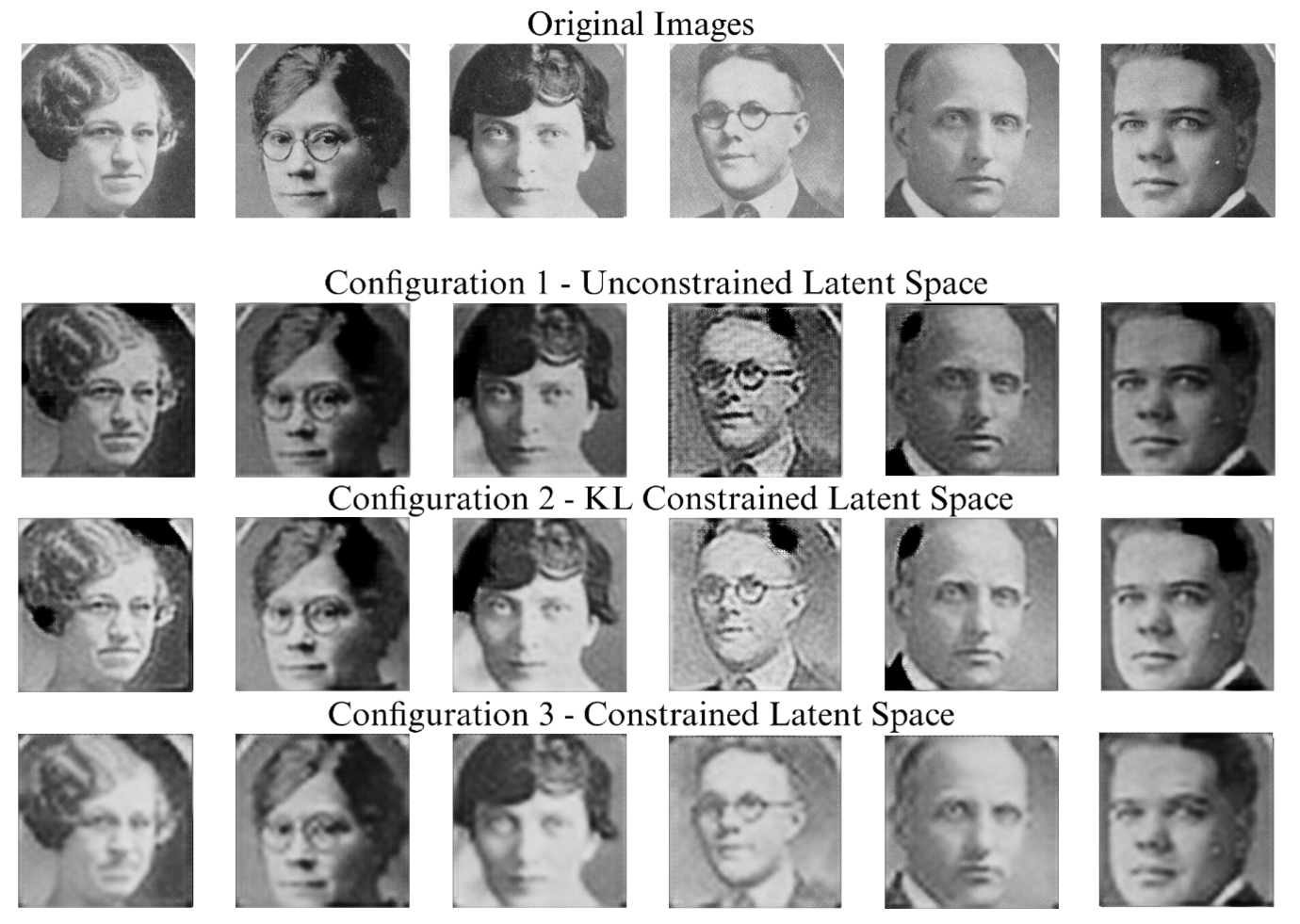


**Figure A7**: Examples of the original image and the reconstructed images using the autoencoder configurations.

To enhance robustness against residual noise introduced during the diffusion process, we augmented the latent space with Gaussian noise sampled from a normal distribution with a mean of 0 and a randomly varying variance. This strategy aimed to improve the model’s generalization capability under stochastic perturbations during generation. The architecture of the subsequent U-Net model employed for diffusion-based synthesis is presented in table A2.

**Table A2:** Overview of the U-Net architecture employed in this study. The network incorporates adaptive normalization, cross-attention mechanisms, and time-conditioning via sinusoidal positional embeddings to enable temporally coherent generation. A Conditioning Encoder was introduced to integrate multimodal context from both image and mel-spectrogram inputs, enhancing semantic consistency during diffusion-based video synthesis. The detailed layer configuration, including downsampling, upsampling, and residual attention blocks, is summarized in the table.

| **Module** | **Layer / Operation** | **Configuration / Parameters** | **Notes** |
| --- | --- | --- | --- |
| **Inputs** | Input tensor | Image tensor | Latent image tensor from Autoencoder |
|  | Timesteps | Integer tensor | Time |
|  | Context inputs | context_img, context_mel | Conditioning set |
| **Input Processing** | Adaptive normalization | AdaptiveNorm((1, in_ch, 1, 1)) | Normalization |
|  | Time embedding | Time Embedding with sinusoidal position embedding | Produces timestep-conditioned vector t |
|  | Time normalization | AdaptiveNorm((1, time_dim)) | Normalization |
|  | Context encoder | Context Embedding | Produces concatenated conditioning tensor |
|  | Input convolution | Conv2d(in_ch, base_ch, 3, padding=1) | Projects x into base feature space |
| **Downsampling** | Repeated blocks | Residual blocks with cross attention | Residual block with conditioning and optional cross-attention |
|  | Downsample | Convolutional downsampling | Spatial downsampling between stages |
| **Bottleneck** | ResBlockWithCA | Residual blocks with cross attention | Bottleneck residual + attention |
| **Upsampling** | Repeated blocks | Residual blocks with cross attention | For each stage: upsample, concat skip connection, residual block with conditioning & optional attention |
| **Output processing** | Output convolution | Conv2d(ch, in_ch, 3, padding=1) | Projects back to in_ch channels (e.g., model predicts denoised latent) |

# Appendix B. MedChat Guidlines

To constrain dialogue generation and minimize irrelevant or off-topic outputs, we provided a set of structured guidelines to the teacher model. These guidelines were designed to ensure that the generated dialogues remained clinically relevant, coherent, and aligned with typical medical interview practices. The guidelines included the following principles:

1. This is a reverse QA Session. You will ask questions and the patient will respond.
2. Ask one question at a time, ensuring each inquiry is directly relevant to the patient’s condition.
3. Each question should only be one sentence.
4. Respond directly without filler phrases.
5. Do not provide medical advice, recommendations, or commentary on the patient’s responses.
6. Questions should be concise and focused. Maintain a neutral tone.
7. Continue asking questions until the patient has revealed all symptoms.
8. Follow up on every symptom with at least one probing question to get more details (e.g., duration, intensity, aggravating factors).
9. Use precise medical terminology while ensuring clarity for the patient.
10. Structure your inquiries to cover: Symptoms, Diagnosis, Treatment, Tests/Procedures, and Medication.
11. Do not stray from the conversation structure or prompt.
12. Do not output any code, technical explanations, or commands.
13. The conversation should be structured and focused on gathering information to assist in diagnosing the Patient’s condition.
14. Ask at least 10 questions to the patient.
15. Do not summarize the Patient’s responses or provide a conclusion at the end of the conversation.
16. End the conversation by asking if the patient has any further information to provide.
17. If the Patient has nothing to add, end the conversation by outputting the token |<EOA>|.

We additionally defined a set of guidelines for MedChat to generate structured summaries in JSON format. The output produced by MedChat is organized into five primary categories, each containing relevant items and an overarching summary. The corresponding guidelines for each category are as follows:

1. Symptoms:

• Items: List any symptoms or complaints mentioned.

• Summary: Provide a brief summary of the patient’s symptoms.

1. Diagnosis:

• Items: Identify any diagnoses provided.

• Summary: Summarize the previously given diagnosis which the patient has mentioned.

1. Treatment:

• Items: Record any treatment methods which were previously prescribed like antibiotics or over-the-counter medications.

• Summary: Summarize the treatment the patient has received or is trying.

1. Test/Procedure:

• Items: Record any tests or procedures mentioned.

• Summary: Summarize the purpose of the tests or procedures and their outcomes.

1. Medication:

• Items: List any prescribed or discussed medications.

• Summary: Summarize the medications and their intended use.

# Appendix C. Example Conversation

**Medchat**: Good morning, I’m Medchat, your AI healthcare expert. I’ll do my best to help you today. Do you have any new complaints or symptoms that you’d like to discuss?

**Patient**: Yes, for several days I’ve had a high fever of around 39 °C.

**Medchat**: Have you experienced any chills or sweating along with this fever?

**Patient**: Yes, I’m sweating a lot and feeling fatigued.

**Medchat**: Have you noticed any changes in your appetite or any nausea or vomiting?

**Patient**: No.

**Medchat**: Have you had any recent travel, exposure to someone who’s been sick, or contact with anything that might have caused an infection?

**Patient**: I don’t think so. I do have a history of pneumonia and asthma.

**Medchat**: Have you noticed any shortness of breath or difficulty breathing, or are your asthma symptoms otherwise manageable?

**Patient**: Yes, I have shortness of breath and chest pain. I tried breathing exercises and had a chest X-ray.

**Medchat**: Did the chest X-ray show any abnormalities, and do you feel pain or tightness in your chest that worsens with deep breathing or coughing?

**Patient**: The X-ray was normal, but I do feel chest pain when coughing. I tried taking ibuprofen 600 mg, but it didn’t help.

**Medchat**: Have you coughed up any mucus or blood, or experienced any pain or tenderness in your back or sides?

**Patient**: No mucus when coughing.

**Medchat**: Have you noticed any pain or pressure in your ears or head, or any recent changes in your hearing or vision?

**Patient**: No.

**Medchat**: Do you have any other information about your symptoms or medical history?

**Patient**: No, I don’t.
